# Comparative Gene Mapping as a Tool to Understand the Evolution of Pest Crop Insect Chromosomes

**DOI:** 10.3390/ijms18091919

**Published:** 2017-09-07

**Authors:** Mauro Mandrioli, Giada Zambonini, Gian Carlo Manicardi

**Affiliations:** Department of Life Sciences, University of Modena and Reggio Emilia, Modena 41125, Italy; mauro.mandrioli@unimore.it (M.M.); giadazambonini@hotmail.it (G.Z.)

**Keywords:** aphid chromosomes, Muller elements, chromosomal rearrangements, synteny

## Abstract

The extent of the conservation of synteny and gene order in aphids has been previously investigated only by comparing a small subset of linkage groups between the pea aphid *Acyrthosiphon pisum* and a few other aphid species. Here we compared the localization of eight *A. pisum* scaffolds (covering more than 5 Mb and 83 genes) in respect to the *Drosophila melanogaster* Muller elements identifying orthologous loci spanning all the four *A. pisum* chromosomes. Comparison of the genetic maps revealed a conserved synteny across different loci suggesting that the study of the fruit fly Muller elements could favour the identification of chromosomal markers useful for the study of chromosomal rearrangements in aphids. *A. pisum* is the first aphid species to have its genome sequenced and the finding that there are several chromosomal regions in synteny between Diptera and Hemiptera indicates that the genomic tools developed in *A. pisum* will be broadly useful not only for the study of other aphids but also for other insect species.

## 1. Introduction

A large number of insect genomes have been wholly sequenced in the last decades in order to better understand their biology and, in particular for pest crop insects, to identify genes that could represent a potential target for their control in the field [1,2,3,4,5,6].

Insects are essential to maintaining agricultural ecosystems, but some of them are pests that damage >30% of agricultural, forestry, and livestock production and cause billions in economic losses annually. Currently, the genomes of at least 140 insects have been sequenced and deposited in public databases and the availability of insect genomes and transcriptomes provided valuable resources for entomological research [1,2,3,4,5,6]. Indeed, insect genomics allowed the gain of knowledge in several fields, such as functional genomics, comparative analysis of genomic contents and their organization, as well as functional analyses of critical parameters as their capacity to transmit disease agents. A better understanding of many individual genes and gene families has been obtained as well [1,2,3,4,5,6]. However, most of these projects (except Diptera) completely lacked any information about the chromosomal localization of the identified genes and, as a consequence, the involvement of chromosomal rearrangements in insect biology has been almost neglected.

Data concerning the chromosomal localization of the annotated genes could be, for instance, extremely relevant to understanding the evolution of the sex chromosomes and the sex determining system, which is a topic of great interest for pest crop insects [7,8].

The genome mapping in Diptera evidenced that in Drosophila species six different chromosome arms, the so-called ‘‘Muller elements,” constitute the building blocks for all Drosophila species. The conservation of the Muller elements extends far beyond Drosophila to, at least, tephritid fruit flies, thought to have diverged from drosophilids 60–70 million years ago, favouring the understanding of the mechanisms that shaped the evolution of the dipteran karyotype [9,10,11,12,13,14,15]. For instance, chromosomal arms exhibit significant remnants of homology between *D. melanogaster* and *Anopheles gambiae*, despite the fact they diverged about 250 million years ago, and about 34% of their genes colocalize in “microsyntenic” clusters [10].

The genome of the aphid *Acyrthosiphon pisum* has been published in 2010 [16] and it favoured a better understanding of the biology of Hemiptera, a taxon consisting of a large number of pest crops species [17,18].

Few studies have been focused on the construction of genetic maps in aphids [19]. The first densest pea aphid genetic map has been developed by Hawthorne and Via [20] with the aim to study the aphid host plant specialization. They developed a linkage map of 173 dominant amplified fragment length polymorphism (AFLP) markers grouped into four linkage groups. Successively, Braendle et al. [21] developed an additional seven AFLP markers on the X chromosome.

From a cytogenetic point of view, aphid chromosomes have been studied mainly in order to identify cytogenetic markers that could be useful for taxonomic identification, as well as for the analysis of karyotype evolution [22,23,24,25,26,27]. At this time, few genes have been located on chromosomes in aphids [28,29].

In order to improve our knowledge about the gene distribution on aphid chromosomes and to suggest a strategy for the identification of chromosomal markers, here we compared the localization and composition of eight scaffolds (spanning 5.3 Mb and 83 genes) identified in *A. pisum* in respect to the fruit fly Muller elements. This approach allowed the identification of orthologous loci spanning all the four *A. pisum* chromosomes. In view of the suggestion that *A. pisum* shows a substantial synteny (together with conserved gene order and orientation) with other Aphidinae [30,31], our approach could be useful to extend genomic information from *A. pisum* to other aphid species. Lastly, comparative mapping can facilitate not only the investigation of specific evolutionary questions, but also the study of synteny at genomic scales to elucidate chromosome homology, providing a framework for predicting the location of genes in other species, including insects of agricultural interest.

## 2. Results

**Figure 1:**
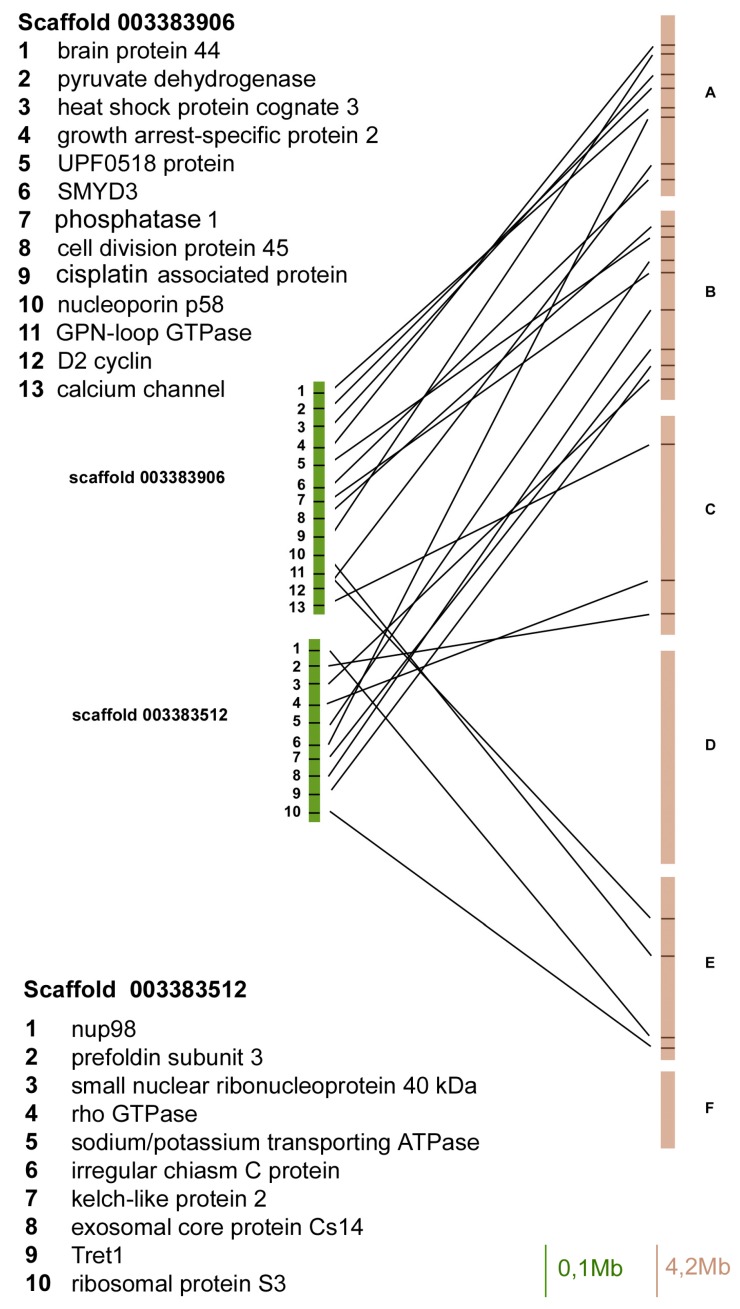
Gene content and reciprocal position of genes mapped in scaffold 003383906 and 003383512 in *Acyrthosiphon pisum* (green) and in *Drosophila melanogaster* (brown) Muller elements.

**Figure 2:**
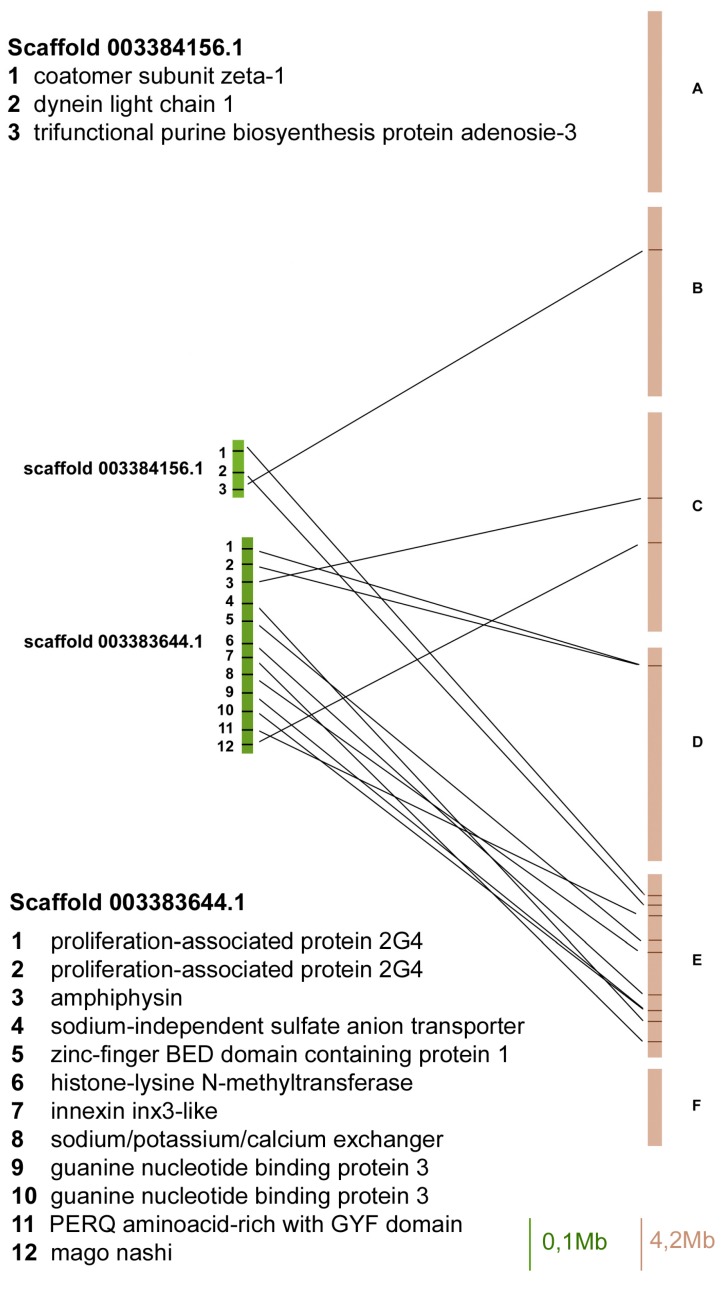
Gene content and reciprocal position of genes mapped in scaffold 003384156 and 003383644 in *A. pisum* (green) and in *D. melanogaster* (brown) Muller elements.

**Figure 3:**
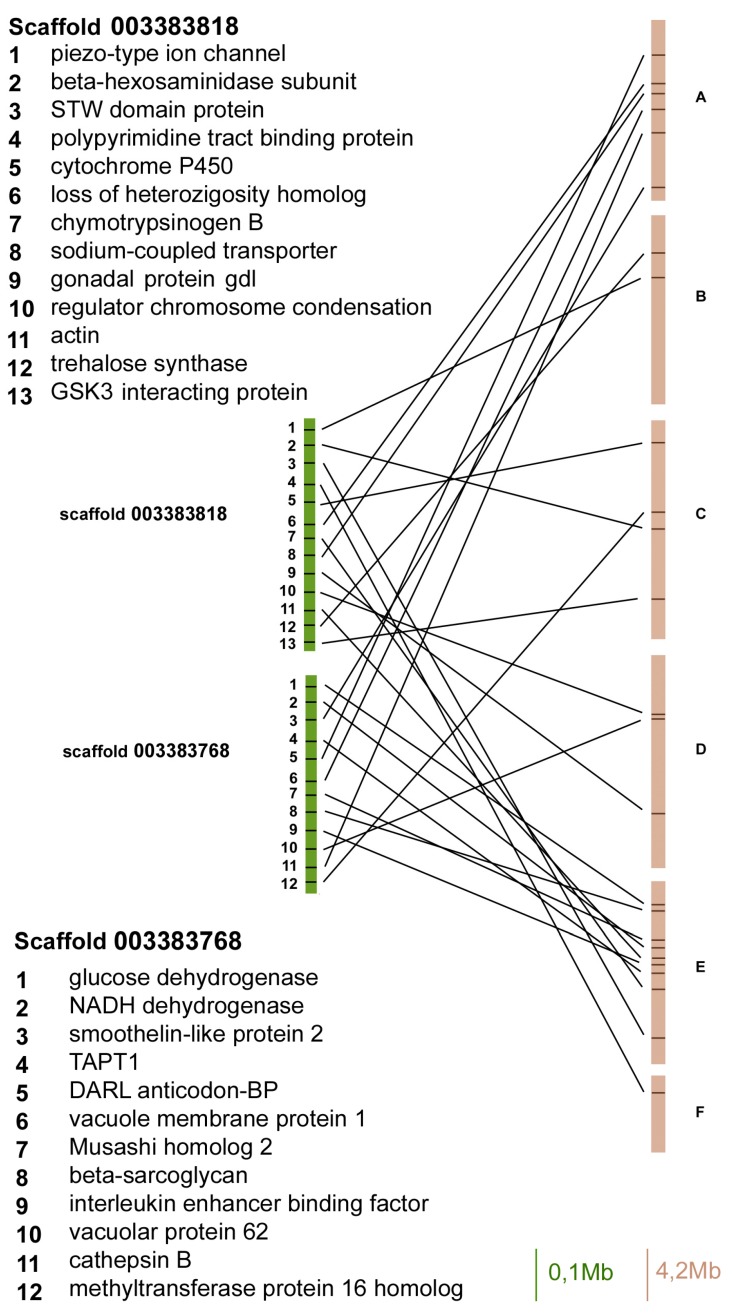
Gene content and reciprocal position of genes mapped in scaffold 003383818 and 003383768 in *A. pisum* (green) and in *D. melanogaster* (brown) Muller elements.

**Figure 4:**
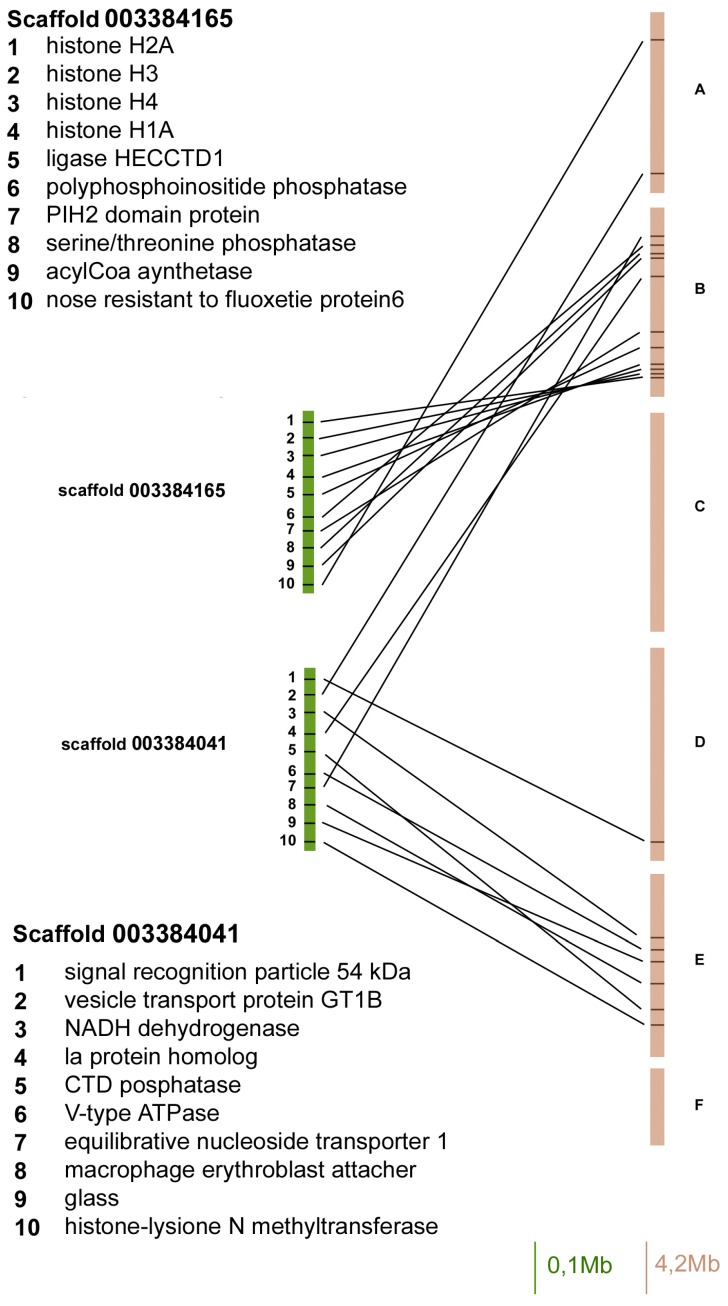
Gene content and reciprocal position of genes mapped in scaffold 003384165 and 003384041 in *A. pisum* (green) and in *D. melanogaster* (brown) Muller elements.

In order to compare the localization of genes between the *A. pisum* genome and the *D. melanogaster* Muller elements, we identified a set of 83 *A. pisum* genes (isolated from 8 scaffolds) with orthologues in the fruit fly genome and verified their localization (Figure 1, Figure 2, Figure 3 and Figure 4).

In particular, in the scaffold 003383906 we identified 13 *A. pisum* genes with orthologues in fruit flies that mapped on Muller elements A, B, C, and D, but 8 of 13 mapped on the Muller element A (Figure 1).

The scaffold 003383512 presented 10 orthologous genes in *A. pisum* and *D. melanogaster*, and five of them mapped on the fruit fly Muller element B, whereas the other ones were located in elements A, C, and E (Figure 1).

The scaffold 003384156 contained only three orthologues and two of them mapped on Muller element E (Figure 2).

The scaffold 003383644 presented 12 orthologous genes and eight of them mapped on Muller element E, whereas the others mapped on D and C elements (Figure 2).

The scaffold 003383818 contained 13 orthologues and three of them mapped on Muller element E, whereas the other ones mapped on A, B, C, and D elements (Figure 3).

The scaffold 003383768 contained 12 orthologous genes between aphids and flies, and six of them mapped on Muller element E, whereas the others mapped on A, C, and D elements (Figure 3).

Both the scaffold 003384165 and 003384041 contained 10 orthologous genes, but 9 out of 10 genes in the scaffold 003384165 mapped on Muller element B, whereas 6 out of 12 of the scaffold 003384041 have been located on element E (the other ones in Muller elements A, B, C, and D).

**Figure 5:**
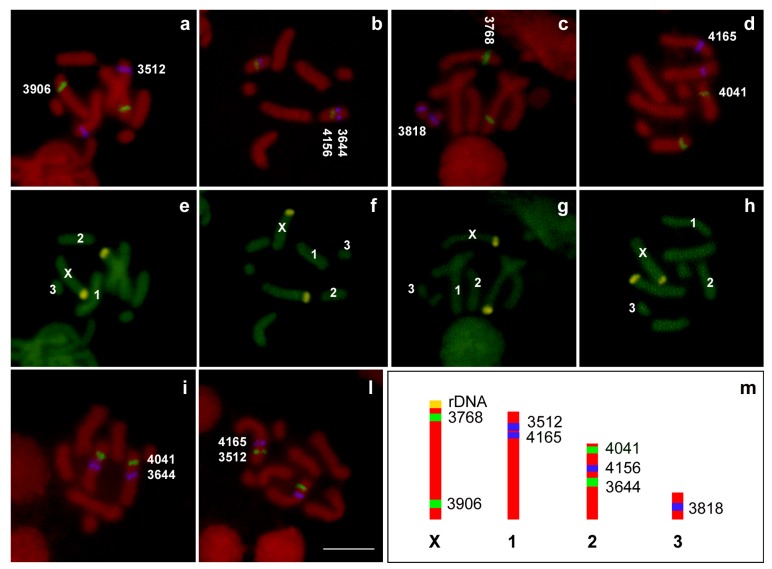
Double fluorescent in situ hybridization (FISH) with fluorescein isothiocyanate (FITC)-labelled 3906 (**a**), 4156 (**b**), 3768 (**c**), and 4041 (**d**) probes (in green) and with aminomethylcoumarin acetate (AMCA)-labelled 3512 (**a**), 3644 (**b**), 3818 (**c**), and 4165 (**d**) probes (in blue) allowed the mapping of the *A. pisum* scaffolds on the chromosomes, counterstained with propidium iodide (in red). In order to define the reciprocal position on chromosomes, double FISH experiments with FITC-labelled 4041 (**i**) and 3512 (**l**), and AMCA-labelled 3644 (**i**) and 4165 (**l**) probes have also been performed. Chromomycin A_3_ staining (**e**–**h**) allowed the identification of the X chromosomes in each plate. A schematic representation (**m**) allowed the comparison of the localization of each scaffold on the *A. pisum* chromosomes. **3906**: scaffold 003383906. **4156**: scaffold 0033844156. **3768**: scaffold 003383768. **4041**: scaffold 003384041. **3512**: scaffold 003383512. **3644**: scaffold 003383644. **3818**: scaffold 003383818. **4165**: scaffold 003384165. Bar corresponds to 100 μm.

The chromosomal localization of the eight *A. pisum* scaffolds has been successively investigated by FISH. As summarized in Figure 5, the scaffolds 003383768 and 003383906 mapped on the opposite telomeres of the X chromosomes, identified since they are the unique ones with a chromomycin A_3_ (CMA_3_)- fluorescent telomere, which is a rule in aphid complements (Figure 5a,c). Fluorescent in situ hybridization (FISH) experiments mapped scaffolds 003383512 and 003384165 in the same half of autosomes 1 (Figure 5a,d) and scaffolds 003384041, 003384156, and 003383644 on autosome 2 (Figure 5b,d). Scaffold 003383818 was the unique one mapping on autosome 3 (Figure 5c).

**Figure 6:**
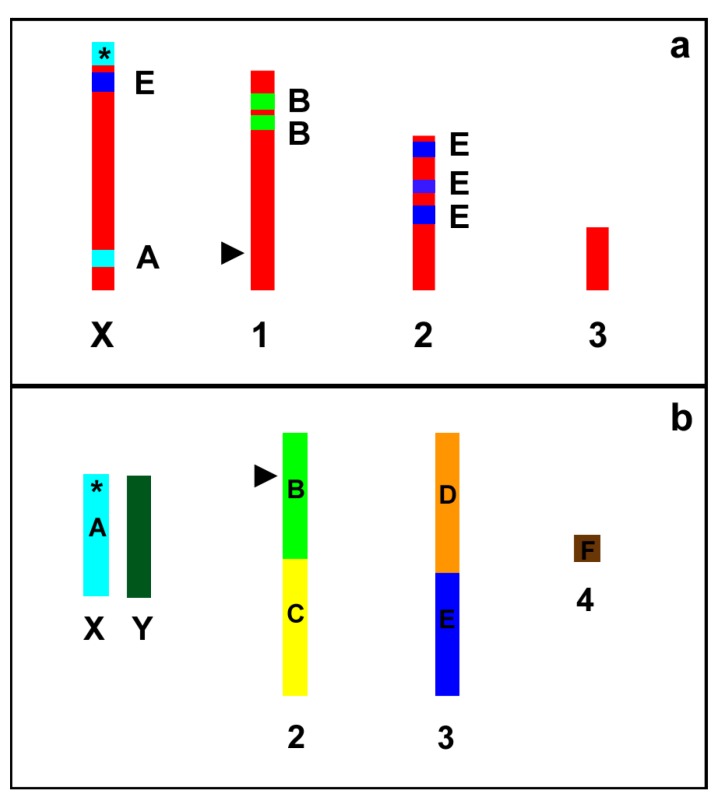
Schematic representation (not to scale) of the *A. pisum* (**a**) and *D. melanogaster* (**b**) karyograms showing the localization of the A–F Muller elements in the fly chromosomes and the trait of synteny with the Muller elements in the aphid chromosomes. Asterisks indicate the localization of the genes coding for ribosomal RNA (rDNA). Arrow heads indicate the position of the genes coding for 5S rRNA.

As a whole, the search of scaffolds with synteny between pea aphids and fruit flies allowed the identification of orthologous loci spanning all the four *A. pisum* chromosomes. Furthermore, our results show that, even if the gene order is highly scrambled, a trace of the Muller elements is present also in aphids (Figure 6).

## 3. Discussion

*D. melanogaster* represents a largely utilized model system for animal and insect genetics [4,32,33,34]. The huge amount of available information from *Drosophila* provides valuable data for the analysis of gene regulation, genetic diseases, and evolutionary processes [4,32,33,34].

Several papers, published more than 70 years ago, evidenced the presence of recurrent traits of homology in the six chromosomal elements within the *Drosophila* genus [9,35]. The conservation of these basic elements, named A–F by Muller [35], has been successively confirmed also in the medfly *Ceratitis* *capitata* [36,37], as well as in the olive fly *Bactrocera* *oleae* [38]. Moreover, the chromosome homology among several *Bactrocera* species and *C*. *capitate*, as well as between *Anastrepha *ludens** and *C*. *capitate*, has been established based on both their polytene chromosome banding pattern similarities and/or in situ hybridization of selected probes [15,39,40,41,42,43].

As reported by Sved et al. [15], Muller elements are not conserved in the *Drosophila* genus only, but also in tephritid fruit flies, even though they diverged from drosophilids about 60–70 mYr ago. According to data on *Drosophila* species, gene order may be highly scrambled within each Muller element, thus indicating high levels of intra-chromosomal rearrangements [15,42,43]. The stability in the gene chromosomal localization observed in Diptera is in stark contrast with data collected in other taxa [15]. Indeed, higher rates of inter-chromosomal rearrangements occurred over comparable time spans in most other groups, such as eutherian mammals [44], cichlid fishes [45], finches [46], and plants [47].

The presence and conservation of the Muller elements have not been studied outside Diptera, despite their potential usefulness to understand the evolution of the insect karyotype and the possibility to favour the development of chromosomal markers also in other insect species.

Although aphid chromosomes have been studied for several decades, at present few genes have been located on chromosomes, other than the 28S rDNA genes located at one telomere of the X chromosomes, and they include the 5S rDNA [24] and histone genes [48] in both *A. pisum* and *M. persicae*, as well as the esterase E4 coding genes in *M. persicae* only [49]. Moreover, ten satellite DNAs have been also identified and localized on chromosomes in five aphid species: one in *Megoura viciae* [50], one in *Rhopalosiphon padi* [51], two in *M. persicae* [22,52], two in *Amphorophora tuberculata* [53], and four in *Aphis nerii* [26].

In the present paper we localized more than 80 genes spanning all the four *A. pisum* chromosomes clearly supporting the proposal that the search for Muller elements could greatly improve the development of chromosome-specific markers in insects outside the *Drosophila* genus. Indeed, our results show that, even if the gene order is highly scrambled (as expected from the literature data reported about Diptera), a trace of the Muller elements is still present in aphids, even if the divergence between *A. pisum* and *D. melanogaster* can be estimated to 320–390 million years ago [54].

The presence of the trait of homologies/synteny between aphids and Diptera is particularly relevant considering that aphids possess holocentric chromosomes with kinetic activity spread along the whole chromosome axis, as well as a reproduction based on apomictic parthenogenesis that could favour the occurrence of chromosomal rearrangements and their inheritance [28,29].

The occurrence of this macrosynteny, combined to the presence of genes differently ordered and distributed on chromosomes, is similar to what was observed in the of holocentric chromosomes of Lepidoptera [55]. Indeed, as reported by d’Alençon et al. [53], a high degree of synteny was present between *Bombyx mori* and two noctuid species even if high rates of local genome rearrangements have been observed. Conserved syntenic blocks of genes were very small in Lepidoptera since they approximately contain 1.3 genes per block between *B. mori* and two noctuid species, and 2.0 genes per block between *Spodoptera frugiperda* and *Helicoverpa armigera* [55]. This corresponds to approximately two chromosome breaks per Mb DNA per million years, which is an evolution rate much higher than among species of the *Drosophila* genus [42,43]. It seems, therefore, that holocentric chromosomes could favour local rearrangement without significantly affecting the synteny at the overall chromosomal level.

A further element that could explain this result is related to the gene density and distribution on chromosomes [28,29]. Even if, at present, there is no detailed information regarding the distribution of genes on arthropod holocentric chromosomes [28,29], previous cytogenetic analyses suggested that, in aphids, the distribution of genes was uniform throughout all autosomes, with some differences related to X chromosomes where a certain degree of compartmentalization has been observed [56]. The occurrence of a uniform gene distribution counteracts data collected in other insects, such as *D. melanogaste* and *Anopheles gambiae*, where three isochore families have been identified with gene density increasing in GC-rich isochores [57] and could be a feature of species with holocentric chromosomes. As a whole, we can suggest that chromosome rearrangements, facilitated by the holocentric nature of chromosomes, disrupted gene-rich chromosomal regions, bringing them to a uniform gene distribution without affecting the overall macrosynteny among aphids and other insects.

Considering that aphids and Lepidoptera share the presence of holocentric chromosomes, it can be suggested that the scattered organization of centromeric determinants (related to their holocentric nature) may lead to a greater genomic plasticity, as chromosome fragments resulting from double-strand breaks can be maintained favouring intra-chromosomal rather than inter-chromosomal rearrangements.

According to literature data [30,31], *A. pisum* show substantial synteny of gene order and orientation with other Aphidinae, with excellent prospects for being able to extend genomic information from *A. pisum* to other aphid species. As a consequence, the study of Muller elements in aphids could favour the identification of the chromosomal marker also in other aphid species, such as *M. persicae*, where several chromosomal rearrangements have been observed, but a full molecular cytogenetics analysis is still lacking in view of the absence of chromosomal markers.

## 4. Materials and Methods

The specimens of the pea aphid, *A. pisum*, used in the present research, were obtained from the LSR1 laboratory lineage, kindly furnished by Manuel Plantagenest (INRA, Le Rheu, France) and maintained asexually on broad bean *Vicia faba* plants at 19 °C at a light-dark regime of 16 h light:8 h darkness. The *A. pisum* LSR1 lineage was used, since it is the pea aphid lineage sequenced for the genome project [16].

Chromosome preparations were obtained from parthenogenetic females by spreading embryo cells, as reported by Mandrioli et al. [22], whereas CMA_3_ staining was done as described by Mandrioli et al. [26].

DNA extraction was done using the Wizard^®^ SV Genomic DNA Purification System (Promega, Madison, WI, USA), according to the manufacturer’s instructions. The Long PCR Enzyme Mix (Fermentas, St. Leon-Rot, Germany), combined to a digoxigenin (DIG)- and biotin-labelling of the probe with the PCR DIG labelling kit (Roche, Sdney, Australia), has been used to amplify and label two contiguous 20 Kbp long probes for each scaffold. The use of two probes for each scaffold has been preferred, since it allows longer labelled chromosomal portions and, consequently, more evident fluorescent signals on chromosomes. Oligonucleotide primers have been specifically designed on the scaffold sequences (Table 1) using the freely available software Primer 3 (available online: http://bioinfo.ut.ee/primer3/).

Fluorescent in situ hybridization (FISH) was performed as described by Mandrioli et al. [26] using fluorescein isothiocyanate (FITC)-conjugated anti-DIG antibodies (Roche, Sidney, Australia) for the DIG labelled probes and aminomethylcoumarin acetate (AMCA) coniugated-avidin for the biotin labelled probes. FISH slides were observed using a Zeiss Axioplan epifluorescence microscope. Photographs of the fluorescent images were taken using a CCD camera (Spot, Digital Instrument, Madison, WI, USA) and the Spot software supplied with the camera and processed using Adobe Photoshop (Adobe Systems, Mountain View, CA, USA).

Bioinformatic analyses were done by BLAST alignments in Genbank (available online: http://blast.ncbi.nlm.nih.gov/Blast.cgi), both at DNA and protein level. Later, a further search was performed by BLAST alignments of aphid genomes using AphidBase (available online: http://www.aphidbase.com). The assembly 2.0 of the pea aphid genome were used for our analyses.

## Figures and Tables

**Table 1:** List of primer for the amplification of the FISH probes.

Scaffold/Probe Name	Primer Name	Primer Sequence
*Scaffold 0033844156* (NW_003384156) probe 1	4156F1	5′-CTTGTATGTTTTGTATGCGTGAGAC-3′
	4156R1	5′-AACAAATTTCAGTTAAACGCAGAAC-3′
*Scaffold 0033844156* (NW_003384156) probe 2	4156F2	5′-TATATGAATAAGCCATGACAAATAA-3′
	4156R2	5′-ATTATGAATATAAAGACGAGCCTAA-3′
*Scaffold 0033833644* (NW_003383644) probe 1	3644F1	5′-TAGGTAGCTGTATAACCCAGTTTCG-3′
	3644R1	5′-AACAGACGGTGTGTAGGTATGGTAT-3′
*Scaffold 0033833644* (NW_003383644) probe 2	3644F2	5′-CAGCATTATACGCATAGGTAGGACT-3′
	3644R2	5′-AAAACTTGTCATGTGTTTTCTGACA-3′
*Scaffold 003383818* (NW_003383818) probe 1	3818F1	5′-TTGTTCTCATTGGATTTATTTGGTT-3′
	3818R1	5′-AAGTGAGGTACTAATTCGTGTCCAG-3′
*Scaffold 0033833818* (NW_003383818) probe 2	3818F2	5′-CTGGACACGAATTAGTACCTCACTT-3′
	3818R2	5′-TTCATTGCATACAAAACATGGTATC-3′
*Scaffold 003383768* (NW_003383768) probe 1	3768F1	5′-TACCAACGTCGTACATACACCATAC-3′
	3768R1	5′-ATTATTGATGCCCATTTTACTACGA-3′
*Scaffold 0033833768* (NW_003383768) probe 2	3768F2	5′-TGGCTATGTGTCGTTATGAATTAGA-3′
	3768R2	5′-CCAAGTTTGTGAAAATGGTTAAATC-3′
*Scaffold 003383906* (NW_003383906) probe 1	3906F1	5′-TAGAAATCAGTGTCATGAAGGATGA-3′
	3906R1	5′-CTAGTCAACACGGGTAATGAGAGTT-3′
*Scaffold 0033833906* (NW_0033838906) probe 2	3906F2	5′-ATCACTCACACATTCGTTTTCAGTA-3′
	3906R2	5′-TTATTTTCCACCACTTTTCAATCAT-3′
*Scaffold 003383512* (NW_003383512) probe 1	3512F1	5′-CGGTATCAGTTCGTTAAGCATAAGT-3′
	3512R1	5′-ATACAATTGATGAATCGGTTGAGTT-3′
*Scaffold 0033833512* (NW_0033838512) probe 2	3512F2	5′-AACCAATACATTCAAGAATTTCCAA-3′
	3512R2	5′-CACACGACGTCATCTAGTACAAATC-3′
*Scaffold 003384165* (NW_003384165) probe 1	4165F1	5′-TTTAATATTGATTGCTCCGTATGGT-3′
	4165R1	5′-TCATTATCCAAAAGAAAGGAGACTG-3′
*Scaffold 003384165* (NW_003384165) probe 2	4165F2	5′-TGATACCGATTGTGATTTTAAGGAT-3′
	4165R2	5′-GTTCAAAGACTGATCGTACATGTTG-3′
*Scaffold 003384041* (NW_003384041) probe 1	4041F1	5′-TTGTACCTGCACATTGTAGACCTAA-3′
	4041R1	5′-ACAACTAACTGCAGGTCTTTATTGG-3′
*Scaffold 003384041* (NW_003384041) probe 2	4041F2	5′-GATTTCTCATTGATACGGCTTCTAA-3′
	4041R2	5′-CCATGGTTTGAGTGTACTTCTTCTT-3′
